# Omega-3 fatty acid intake and prevalent respiratory symptoms among U.S. adults with COPD

**DOI:** 10.1186/s12890-019-0852-4

**Published:** 2019-05-21

**Authors:** Chantal M. Lemoine S, Emily P. Brigham, Han Woo, Corrine K. Hanson, Meredith C. McCormack, Abigail Koch, Nirupama Putcha, Nadia N. Hansel

**Affiliations:** 1 Johns Hopkins University School of Medicine, Baltimore, MD USA; 2 University of Nebraska Medical Center, Omaha, NE USA; 3 Johns Hopkins University Bloomberg School of Public Health, Baltimore, MD USA

**Keywords:** Omega, Fatty acid, COPD, Education, Smoking

## Abstract

**Background:**

Omega-3 fatty acids, including alpha-linolenic acid (ALA), eicosapentaenoic acid (EPA), docosahexaenoic acid (DHA) and derivatives, play a key role in the resolution of inflammation. Higher intake has been linked to decreased morbidity in several diseases, though effects on respiratory diseases like COPD are understudied.

**Methods:**

The National Health and Nutrition Examination Survey (NHANES), with a focus on dietary assessment, provides a unique opportunity to explore relationships between omega-3 intake and morbidity in respiratory diseases marked by inflammation in the United States (US) population. We investigated relationships between ALA or EPA + DHA intake and respiratory symptoms among US adults with COPD, as well as variation in relationships based on personal characteristics or exposures.

**Results:**

Of 878 participants, mean age was 60.6 years, 48% were current smokers, and 68% completed high school. Omega-3 intake was, 1.71 ± 0.89 g (ALA), and 0.11 ± 0.21 g (EPA + DHA). Logistic regression models, adjusting for age, gender, race, body mass index, FEV_1_, education, smoking status, pack-years, total caloric intake, and omega-6 (linoleic acid, LA) intake demonstrated no primary associations between omega-3 intake and respiratory symptoms. Interaction terms were used to determine potential modification of relationships by personal characteristics (race, gender, education) or exposures (LA intake, smoking status), demonstrating that at lower levels of LA intake, increasing ALA intake was associated with reduced odds of chronic cough (p_int_ = 0.015) and wheeze (p_int_ = 0.037). EPA + DHA, but not ALA, was associated with reduced symptoms only among current smokers who did not complete high school.

**Conclusions:**

Individual factors should be taken into consideration when studying the association of fatty acid intake on respiratory diseases, as differential responses may reveal susceptible subgroups.

**Electronic supplementary material:**

The online version of this article (10.1186/s12890-019-0852-4) contains supplementary material, which is available to authorized users.

## Background

Chronic obstructive pulmonary disease (COPD) development and morbidity is driven by a combination of host factors and exposures, which increase inflammation in the lung [1]. As a result, patients develop irreversible airflow obstruction and a variety of symptoms, including cough, wheeze, and phlegm production. While a large body of research focuses on environmental exposures as risk factors for disease morbidity, dietary exposures represent an emerging and increasingly recognized contributor to individual respiratory health [2].

Within the broader context of diet, biologically-active fatty acids are hypothesized to play an important role in potentiation and resolution of inflammation, both systemically and in the lung [3, 4]. Omega-3 fatty acids, both plant-derived (α-linolenic acid, ALA) and forms found primarily in fish (eicosapentaenoic acid, EPA and docosahexaenoic acid, DHA) [5] give rise to anti-inflammatory, pro-resolving mediators such as protectins, resolvins, and maresins and protect against pro-inflammatory stimuli [6]. In contrast, omega-6 fatty acids, such as linoleic acid (LA) found in vegetable oils, nuts and seeds are precursors of pro-inflammatory eicosanoids and leukotrienes, and may counterbalance the beneficial effects of omega-3 intake [7]. Though literature links higher dietary intake of omega-3 fatty acids to decreases in cardiovascular risk/atherosclerosis [8], autoimmune disease activity [9] and morbidity in asthma [10], few studies have addressed the effects of omega-3 intake on COPD disease morbidity [11, 12], and fewer account for intake of omega-6 [3].

Using a nationally-representative sample from the US population (the National Health and Nutrition Examination Survey, NHANES) we investigated the relationship between omega-3 fatty acid intake and respiratory symptoms among individuals with COPD, accounting for omega-6 fatty acid intake. Furthermore, given a potential role for unique responses based on participant characteristics or individual exposures, we considered whether race, gender, and socioeconomic status, and additionally smoking status modified the relationship between fatty acid intake and respiratory outcomes.

## Methods

### Study population

The continuous NHANES is a large, nationally-representative, cross-sectional survey administered by the National Center for Health Statistics designed to evaluate the health and nutrition of the U.S. population. The methodology has been published previously [13], approved by the NCHS Research Ethics Review Board, and all study participants gave consent for participation.

Out of 27,528 total participants in the 2007–2012 survey (dates chosen based on availability of spirometry data), the following inclusion and exclusion criteria were defined to isolate individuals with COPD [14]: age 40 years or greater, 100 or more cigarettes smoked lifetime, and pre-bronchodilator FEV_1_/FVC ratio < 0.70 with no asthma diagnosis (see Additional file 1: Figure S1). Furthermore, subjects with energy intake outside a plausible range (≥6000 kcal/day) were excluded [15], for a final sample population of 878.

### Exposures

All included participants completed an initial 24-h dietary recall (all foods and beverages consumed in the prior 24 h) in person at the time of examination, using the Automated Multiple Pass Method from the US Department of Agriculture [16]. Further details can be found in the publicly-available NHANES procedure manuals [13]. The majority (*n* = 796) were reached for a second, telephone-based 24 h recall three to ten days after the exam. Intake of two omega-3 fatty acid groupings (primarily plant-based: ALA, and primarily fish-based: EPA + DHA, g) and omega-6 (linoleic acid, LA) were derived from 24-h recall data, using an average value for participants with two days of data per previous methods [17] and a single value for the minority of participants with one day of data (*n* = 82, < 10% of the sample).

Covariates included age (continuous, years), gender, self-reported race and ethnicity (non-Hispanic white versus others), education (High school graduate and above versus less than high school), smoking status (current versus former), total caloric intake (continuous, kcal), body mass index (BMI, continuous, kg/m^2^), pack-years (continuous), pre-bronchodilator FEV_1_ (continuous, L), and omega-6 fatty acid intake (linoleic acid, LA: continuous, g). Spirometry was performed according to American Thoracic Society guidelines [18, 19]. Participants were notably excluded from spirometry in NHANES based on current chest pain, a physical problem with forceful expiration, or use of supplemental oxygen [13].

### Outcomes

Trained interviewers assessed respiratory symptoms, which were modeled as binary outcomes (yes versus no) based on questions noted in Additional file 2: Table S1 chronic cough, nocturnal cough, phlegm, wheeze (any), nocturnal wheeze, wheeze with exertion, prescription medication use for wheeze.

### Analyses

Sampling weights and strata were used to account for the complex sample design of NHANES, generating estimates representative of the U.S. population. Descriptive statistics were created using survey-weighted means and proportions, with distributions assessed via histogram.

To determine the relationship between omega-3 (ALA or EPA + DHA) intake and symptoms, logistic regression models were created adjusting for participant characteristics including age, gender, race, BMI, FEV_1_, and education, and individual exposures including smoking status, pack-years, total caloric intake, and reported omega-6 (LA) intake.

To determine whether the relationship between omega-3 (ALA or EPA + DHA) intake and symptoms varied by intake of omega-6 (LA), two-way interactions between each omega-3 and omega-6 were evaluated. We also explored whether the relationship between omega-3 (ALA or EPA + DHA) intake varied by participant characteristics including race, gender, and education level in 2-way interaction models, generating omega-3 effects at different levels of each characteristic. Finally, we modeled the modifying effects of smoking status on each of these interactions (3-way interaction models), to determine if the effects of omega-3 intake would differ within participant groups by the presence or absence of a known pro-inflammatory stimulus.

In sensitivity analyses, time spent in sedentary behavior (minutes, continuous) and presence of a relevant comorbidity (dichotomous, including self-reported diagnosis of heart attack, heart failure, heart disease, angina, stroke, cancer, diabetes, hypertension, or hypercholesterolemia) were added to models as confounders. Results did not appreciably change, and these variables are not presented in the final models. Given NHANES exclusion criteria for spirometry, few individuals with GOLD stage III (FEV_1_ 30–49, 6.5% of the sample) and GOLD stage IV (FEV_1_ < 30, 0.2% of the sample) were available for analyses, and sensitivity analyses were not attempted via severity classification.

Modeling assumptions were tested using standard procedures for survey-weighted data, and model fit was evaluated using Wald tests. While statistical significance was defined as *p* < 0.05, given multiple comparisons in interaction models, outcome measures were examined for the emergence of consistent patterns and interpreted in this context. As an additional measure to test statistical significance in the setting of multiple comparisons, analyses were tested while controlling for false discovery rate [20] at 0.2. All analyses were performed using Stata MP Version 15.

## Results

Mean age was 60.6 years, and participants were predominantly male (63.5%) and non-Hispanic white (85.1%) with an average BMI in the overweight range (27.3 kg/m^2^) (Table 1). Average pack-years of smoking were 30 ± 24 and almost half (48%) were current smokers. Over two-thirds (68%) completed at least a high school education. All patients demonstrated obstruction on spirometry per inclusion criteria, with mean + SD FEV_1_/FVC ratio of 0.63 + 0.06 and FEV_1_ of 2.5 ± 0.7 L. Mean + SD energy intake was 2112 + 680 kcal. Average EPA + DHA intake (mean + SD; 0.11 ± 0.21 g; females 0.1 ± 0.1 g and males 0.12 ± 0.3 g), reflected overall low EPA + DHA consumption compared to a recommended intake value of > 0.5 g/day quoted by the American Dietetic Association (ADA) [21] (see Additional file 3: Figure S2 Additional file 5: Table S3). ALA intake (mean + SD; 1.71 ± 0.89 g; females 1.5 ± 0.7 g and males 1.8 ± 1.0 g) was overall higher than ADA adequate intake values of 1.1 g/day (females) and 1.6 g/day (males) [21] (see Additional file 3: Figure S2). Linoleic acid (LA) consumption (mean + SD; 16.5 ± 8.0 g; females 14.4 ± 5.7 g and males 17.8 ± 9.1 g) was within ADA recommended intake levels of 12 g/day (females) and 17 g/day (males) [22]. Wheeze (21%), chronic cough (19.2%) and phlegm (17.7%) were the most prevalent symptoms in the population. Other than by gender, fatty acid intake did not differ significantly by major demographic or exposure characteristics with the exception of EPA + DHA and decile of age (lower intake in the age 40–49 age group Additional file 5: Table S3).

**Table 1:** Characteristics of U.S. Adults with COPD, NHANES 2007–2012

Demographics	
Age (years)	60.6 ± 8.4
Male (%)	63.5
Ethnicity (%)	
Non-hispanic White	85.1
Others	14.8
Pack-Years	30.4 ± 23.8
BMI, kg/m2	27.3 ± 4.9
Dietary Intake	
Energy (kcal)	2112 ± 680
Omega-3	
EPA + DHA (g)	0.11 ± 0.21
ALA (g)	1.71 ± 0.89
Omega-6	
LA (g)	16.5 ± 8.0
Lung Function	
FEV1, L	2.5 ± 0.7
FVC, L	3.9 ± 0.9
FEV1/FVC	0.63 ± 0.06
Symptom Prevalence (%)	
Chronic cough	19.2
Nocturnal cough	6.9
Phlegm	17.7
Wheeze (any)	21.0
Nocturnal Wheeze	9.4
Wheeze with exertion	8.1
Meds for wheeze	5.4

Mean ± SD unless otherwise noted

*n* = 878

BMI: Body Mass Index; EPA: eicosapentaenoic acid; DHA: docosahexaenoic acid; ALA: alpha-linolenic acid; LA: linoleic acid; FEV1: forced expiratory volume in one second; FVC: forced vital capacity

Fully-adjusted logistic regression models, containing one of the omega-3 intake variables (EPA + DHA or ALA) and adjusted for omega-6 (LA) intake did not reveal consistent, statistically significant primary effects of EPA + DHA or ALA intake on respiratory symptoms. A significant association was found between higher ALA and reduced odds of prescription medication use for wheeze (OR 0.43 per 1 g increase in ALA, *p* = 0.015).

**Fig. 1:**
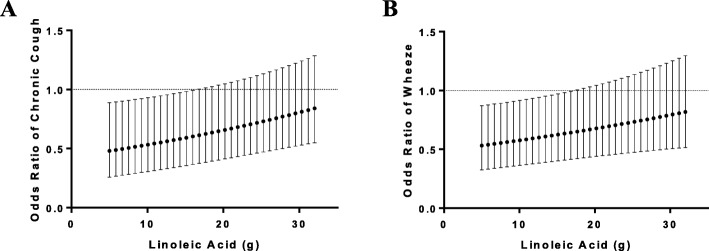
Relationship between ALA Intake and Odds of Respiratory Symptoms is Modified by LA Intake in U.S. Adults with COPD. Odds of chronic cough (Panel A) and wheeze (Panel B) per 1 g increase in ALA. ALA and LA included simultaneously in logistic regression models adjusting for age, gender, race, education, smoking status, FEV_1_, caloric intake, BMI, and pack-years

Adjusted models examining continuous interactions between each omega-3 variable (EPA + DHA or ALA) and omega-6 (LA) intake demonstrated significant changes in the relationship between ALA and several respiratory symptoms by reported intake of omega-6, such that protective effects of increasing ALA intake were only evident at low levels of LA intake (Table 2). Significant interactions were noted for chronic cough, any wheeze and nocturnal wheeze (all p_int_ < 0.05), and the interactions remained statistically significant for cough and any wheeze after evaluation for multiple comparisons via false discovery rate methods. As an example of the effect, at the mean value of LA, U.S. adults with COPD demonstrated a 40% reduction in the odds of cough (*p* = 0.045) and 37% reduction in the odds of wheeze (*p* = 0.039). At higher levels of LA (above 15.5 g) these favorable associations were no longer statistically significant (Fig. 1). No significant interactions were noted between EPA + DHA and LA for any of the outcomes examined (data not shown).

**Table 2:** Relationship between Dietary Intake of Fatty Acids and Respiratory Symptoms in U.S. Adults with COPD

	Alpha-linolenic acid (ALA)		Linoleic Acid (LA)		Interaction (ALA*LA)	
	OR (95% CI)	p-val	OR (95% CI)	p-val	OR (95% CI)	p_int_
Chronic Cough	0.60 (0.36-0.99)	0.045	1.005 (0.97-1.04)	0.781	1.02 (1.00-1.04)	0.015
Nocturnal Cough	0.84 (0.42-1.69)	0.628	1.040 (0.96-1.13)	0.353	1.02 (1.00-1.03)	0.053
Phlegm	0.83 (0.51-1.35)	0.437	1.000 (0.95-1.05)	1.000	1.01 (0.99-1.03)	0.096
Wheeze (any)	0.63 (0.41-0.98)	0.039	1.014 (0.97-1.06)	0.510	1.02 (1.00-1.03)	0.037
Nocturnal Wheeze	0.78 (0.42-1.44)	0.420	1.003 (0.95-1.06)	0.921	1.02 (1.00-1.03)	0.032
Wheeze with Exertion	0.81 (0.38-1.69)	0.563	1.003 (0.95-1.06)	0.916	1.02 (0.99-1.04)	0.106
Meds for Wheeze	0.46 (0.22-0.94)	0.034	1.089 (1.02-1.16)	0.008	0.99 (0.96-1.03)	0.729

Adjusted for age, gender, race, education, smoking status, FEV_1_, total calories, BMI, pack per years ALA and LA levels are centered at mean levels for the NHANES population (age 40–79)

**Fig. 2:**
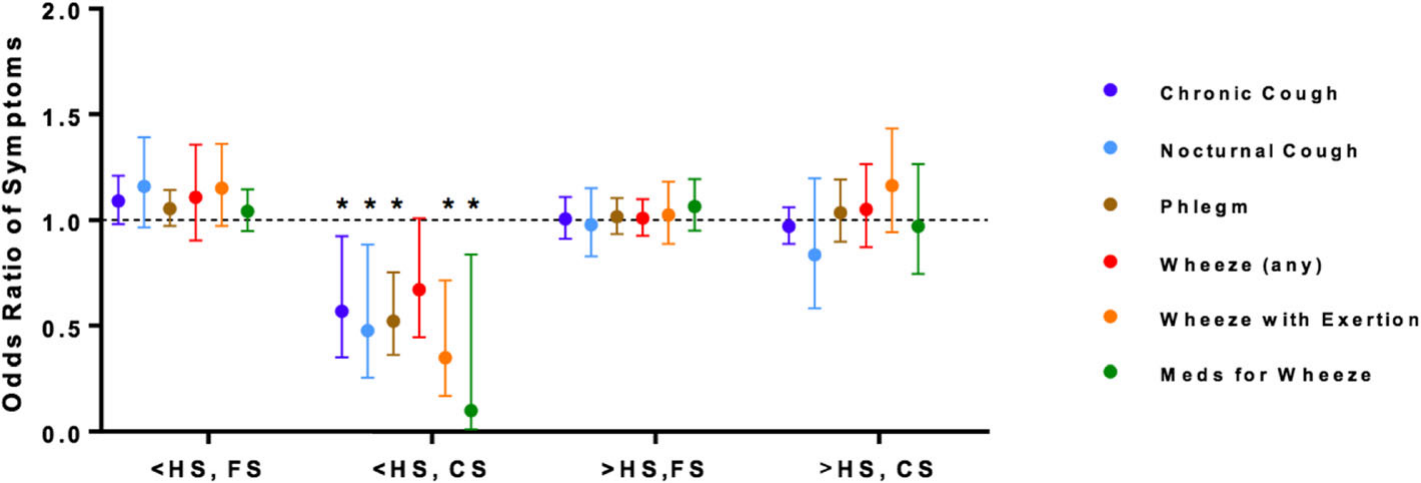
Relationship between EPA + DHA Intake and Respiratory Symptoms is Modified by Socioeconomic and Smoking Status Among U.S. Adults with COPD. 3-way interaction analyses between education, smoking status, and EPA + DHA. Symptoms with a significant interaction are shown (nocturnal wheeze excluded, p_intx_ = 0.767). Logistic regression models adjusted for age, gender, race, FEV_1_, caloric intake, BMI, pack-years, and omega-6 (LA) intake. (<high school, former smoker: *n* = 133; <high school, current smoker: *n* = 149; >high school, former smoker, *n* = 323; >high school, current smoker, *n* = 273) *denotes statistical significance within stratified analyses for *p* < 0.05

Adjusted models examining the potential modifying effects of individual characteristics of race, gender, and education status on the relationships EPA + DHA and ALA on symptom outcomes did not reveal statistically significant associations. However, upon application of smoking status to interaction terms (e.g. 3-way interaction model based on individual characteristic*smoking status*omega-3), a consistent, beneficial effect of increasing EPA + DHA intake was found for respiratory symptoms among current smokers with less than a high school education (Fig. 2; stratified population characteristics, see Additional file 4: Table S2). Significant 3-way interactions were noted for the outcomes of chronic cough (p_int_ = 0.039), nocturnal cough (p_int_ = 0.018), phlegm (p_int_ < 0.001), any wheeze (p_int_ = 0.010), wheeze with exertion (p_int_ < 0.001) and prescription medication use for wheeze (p_int_ = 0.035), and remained statistically significant after controlling for false discovery rate at 0.2 for multiple comparisons. In stratified analyses, within the subgroup of individuals who were current smokers with a less than high school education (n = 149), higher levels of EPA + DHA intake was associated with lower odds of chronic cough (OR = 0.57, *p* = 0.024), nocturnal cough (OR = 0.48, *p* = 0.020), phlegm (0.52, *p* = 0.001), wheeze with exertion (OR = 0.35, *p* = 0.005) and prescription medication use for wheeze (OR = 0.10, *p* = 0.034). No consistent, significant effects were noted with ALA intake, or for other 3-way interaction terms (individual characteristics*smoking status*omega-3) including race or gender (data not shown).

## Discussion

Analyses presented here within a national cohort of adults with COPD demonstrate unique associations between omega-3 intake and respiratory symptoms within susceptible subgroups. Specifically, increased intake of omega-3 (ALA or EPA + DHA) were associated with reduced respiratory symptom prevalence among individuals with lower omega-6 (LA) intake and current smokers with lower education/socioeconomic status. These results are thought provoking and highlight several important themes in a growing body of research linking dietary intake to respiratory health.

Diet is a complex exposure. Previous investigations have traditionally applied one of two approaches to model dietary intake, either focusing on dietary patterns/quality with a combination of nutrients, or concentrating on individual micronutrients within the diet. Omega-3 fatty acids have emerged as prominent within each of these approaches. For example, a “healthy” diet as defined by a Mediterranean-like or Prudent diet, has been linked with lower incidence and prevalence of COPD [22–26], and lower morbidity among individuals with COPD [27]. These diets are traditionally high in omega-3 fatty acids (both plant and fish-derived). Epidemiologic studies focusing on the effects of omega-3 fatty acids have demonstrated some mixed results, but several link increased intake of ALA, EPA, and DHA with lower incidence and prevalence of COPD [28], and decreased inflammatory markers in the serum of adults with COPD [3]. As such, some have proposed omega-3 as a potential “nutraceutical,” or food with medicinal properties that may yield beneficial health effects in COPD and other chronic diseases [29–31].

There is strong rationale for a protective effect of omega-3 fatty acids in the lungs, critical in inflammatory lung diseases such as COPD and supporting plausibility of results. Downstream products of EPA and DHA include specialized pro-resolving mediators, found within the lung and circulation [6] which have the ability to promote resolution of inflammation [32, 33]. These include resolvins, protectins, and maresins, which work by regulating neutrophil infiltration, cytokine production, and macrophage clearance of apoptosed inflammatory leukocytes, culminating in dampening and resolution of inflammatory response [6, 34]. Less is known about the mechanisms by which ALA reduces inflammation. While a variable and limited quantity of ALA is metabolized to EPA and DHA (< 10% each) [35], preliminary work has demonstrated ALA’s ability to inhibit nitric oxide production and TNF-alpha gene expression stimulated by lipopolysaccharide in murine macrophages [36] distinct from the mechanisms of EPA and DHA [37]. Based on this biologic rationale, combined with epidemiologic evidence suggestive of an effect, several trials are underway [38, 39].

Given the anti-inflammatory mechanism by which omega-3 fatty acids are thought to act in the lungs, it is reasonable to postulate that distinct subgroups of individuals, with unique inflammatory responses or distinct exposures linked to inflammation, may respond differently to omega-3 intake. In support of this, we noted that beneficial associations between ALA and respiratory symptoms were only present among those with low omega-6 (LA) intake. This is a reasonable contingency, given: (1) conversion of ALA to EPA + DHA may be inhibited by increased concentrations of LA [40], and (2) LA and its metabolite arachidonic acid are known substrates for production of pro-inflammatory eicosanoids [41], which may counteract the anti-inflammatory properties of ALA.

Personal characteristics may also be important in defining distinct subgroups with unique response profiles. Prior studies have demonstrated that the effects of diet may differ by race [42, 43], gender [44, 45], and socioeconomic status [46]. Each of these participant characteristics is further associated with unique immune responses. Racial and gender differences in immune responses are recognized [47, 48]. Socioeconomic status is linked with access to health care and adherence to medical therapies [49–52] with the potential to reduce local and systemic inflammation. Furthermore, smoking is a potent exposure: dietary effects on respiratory health have been demonstrated to vary by smoking status [25, 53], and exposures to secondhand smoke as well as other forms of air pollution may vary by socioeconomic status [54, 55]. Given the extensive characterization of individuals within the NHANES study, we were able to provide valuable insights into subgroups defined by these characteristics and exposures that may be uniquely affected by EPA + DHA intake.

The weaknesses of our study warrant consideration. Our study population with COPD was defined based on pre-bronchodilator spirometry, and it is possible that some individuals may have been excluded from the analysis with use of post-bronchodilator spirometry. However, pre-bronchodilator spirometry is an acceptable method, widely used in epidemiologic studies in COPD and comparable to definitions previously used in NHANES [14]. This study used a fixed FEV_1_/FVC ratio of 0.7 to define obstruction and COPD, and alternate criteria using characteristics such as gender, age, and height to produce more personalized cutpoints have been proposed [56, 57]. Compared to these criteria, the fixed ratio generally presents a higher specificity for obstruction in younger populations but a higher sensitivity in older populations [58] and has been demonstrated to have clinical relevance in terms of capturing those at higher risk of hospitalization and mortality [59]. Third, few individuals with moderate or severe COPD by GOLD FEV_1_ criteria were included in the analysis, likely in the setting of exclusion from spirometry by NHANES protocol. This limits the potential applicability of findings to the subgroup with mild to moderate disease.

Regarding participant characterization and exposure assessment, our primary exposure (omega-3 intake) was obtained via 24-h recall, inherently subject to both recall bias and possibly information bias as short term intake cannot perfectly approximate long-term or usual nutrient intakes. However, 24-h recall dietary assessment has demonstrated validity in population-based analyses and is consistently used in large epidemiologic studies of dietary exposures [15, 17, 60]. While some individuals only had one 24-h recall available for analysis (< 10% of the sample), use of a single recall is acceptable to assessing the average intake of a population in epidemiologic studies [61]. Representation of socioeconomic status is complex, and education may not capture all aspects; however, education is an established correlate and has been associated with COPD morbidity and mortality [62, 63]. Previous studies have concluded that education as a surrogate of socioeconomic status is the strongest predictor of good health [64].

Analytically, owing to sample size and the complexity of medications available for COPD and other comorbid conditions we were unable to account for the potential effects of pharmacologic therapies with anti-inflammatory properties on the relationship between fatty acid intake and symptoms in this population. Furthermore, multiple relationships between exposures and outcomes were analyzed, increasing the likelihood of a type I error. However, consistency of effect among distinct symptom outcomes strengthens positive findings, and use of false discovery rate methodology reinforces validity.

Despite these limitations, our study has important strengths. NHANES is a nationally representative dataset, and our findings can therefore be extrapolated to the U.S. adult population with COPD. The extensive characterization of the cohort permitted examination of multiple subgroups and identification of characteristics associated with receptivity to beneficial effects of omega-3 intake: low omega-6 intake, and low socioeconomic status and current smokers. We further demonstrated a lack of primary associations between ALA or EPA + DHA in the absence of accounting for omega-6 intake, and did not observe effect modification by race or gender. EPA + DHA intake levels were low compared to ADA recommendations, which may have influenced the ability to detect associations. However, the lack of associations in these subgroups in a nationally-representative cohort are an important aspect of our findings, as they may help to focus future investigations. Additionally, we were able to include ALA and EPA + DHA as individual exposures, key for any future dietary or supplement interventions, and dissect unique relationships with COPD symptom outcomes within each form of omega-3. While the causal impact of a diet high in omega-3 cannot be established from the present observational study, this work reinforces the need for additional investigations, including more objective measurements such as biomarkers, and adds to the evidence base supporting clinical trials of dietary change in inflammatory lung diseases including COPD.

## Conclusions

Within a U.S. population with COPD, we demonstrated associations between ALA intake and lower odds of respiratory symptoms among individuals with average or lower than average intake of pro-inflammatory omega-6. Further, EPA + DHA intake demonstrated a protective effect on respiratory symptoms in subgroups exposed to a myriad of non-dietary inflammatory hazards, specifically, current smokers from low socioeconomic status. A clear understanding of the dietary effects of omega-3 on respiratory outcomes in COPD is yet to emerge; however, the context in which these effects are studied will be an important consideration. Personal characteristics and environmental exposures may be critical to identifying individuals and subpopulations with the greatest opportunity for benefit.

## Additional files

Additional file 1: ** Figure S1.** STROBE Diagram. sample breakdown according to inclusion and exclusion criteria (DOCX 68 kb)

Additional file 2: **Table S1.** Symptom Outcome Questions from the National Health and Nutrition Examination Survey. (DOCX 63 kb)

Additional file 3: ** Figure S2.** Population Distribution of Omega-3 and Omega-6 Fatty Acid Intake in U.S. Adults with COPD. Histograms representing the population distribution of Alpha-linolenic Acid (ALA, omega-3; Panel A, female; Panel B, male) and Eicosapentaenoic Acid + Docosahexaenoic Acid (EPA + DHA, omega-3; Panel C) in grams in U.S. adults with COPD. Vertical red lines represent American Dietetic Association recommended daily intake levels. (DOCX 59 kb)

Additional file 4: ** Table S2.** Stratified Characteristics of U.S. Adults with COPD, NHANES 2007–2012. (DOCX 23 kb)

Additional file 5: ** Table S3.** Intake of omega-3 and -6 fatty acids in U.S. adults with COPD, NHANES 2007–2012. Average fatty acid intake in US/ Individuals with COPD stratified by personal characteristic or exposure. (DOCX 28 kb)

## Abbreviations

ADA: American Dietetic Association
ALA: Alpha-linolenic acid
BMI: Body Mass Index
COPD: Chronic Obstructive Pulmonary Disease
DHA: Docosahexaenoic acid
EPA: Eicosapentaenoic acid
FEV_1_: Forced expiratory volume in one second
FVC: Forced vital capacity
LA: Linoleic acid
NCHS: National Center for Health Statistics
NHANES: The National Health and Nutrition Examination Survey
SD: Standard deviation
US: United States
