# Supplementary material for: Omega-3 fatty acid intake and prevalent respiratory symptoms among U.S. adults with COPD
Source: BMC Pulm Med. 2019 May 21;19:97. doi: 10.1186/s12890-019-0852-4 (PMC6533751; doi:10.1186/s12890-019-0852-4)
Supplement: Supplementary file 1 — Figure S1. STROBE Diagram. sample breakdown according to inclusion and exclusion criteria (DOCX 68 kb) [file 12890_2019_852_MOESM1_ESM.docx]

<100 Cigarettes Lifetime

n= 418

NHANES Continuous

(2007-2012)

n=27,528

n= 9,580

Age<40 years or >79 years

n= 17,948

FEV_1_/FVC ratio <0.70

n= 6,480

n=1,502

n=1,084

Ever Diagnosed with Asthma

n=203

n=881

Daily Caloric Intake >6000 kcal

n=3

Final Analysis

n=878

**Supplemental Figure 1, Additional file 1: STROBE Diagram**
