# Supplementary material for: Omega-3 fatty acid intake and prevalent respiratory symptoms among U.S. adults with COPD
Source: BMC Pulm Med. 2019 May 21;19:97. doi: 10.1186/s12890-019-0852-4 (PMC6533751; doi:10.1186/s12890-019-0852-4)
Supplement: Supplementary file 2 — Table S1. Symptom Outcome Questions from the National Health and Nutrition Examination Survey. (DOCX 63 kb) [file 12890_2019_852_MOESM2_ESM.docx]

**Supplemental table 1, Additional file 2: Symptom Outcome Questions from the National Health and Nutrition Examination Survey**

| Symptom Outcome | Question |
| --- | --- |
| Chronic cough | Do you usually cough on most days for 3 consecutive months or more during the year? |
| Nocturnal cough | In the past 12 months, have you had a dry cough at night not counting a cough associated with a cold or chest infection lasting 14 days or more? |
| Phlegm | Do you bring up phlegm on most days for 3 consecutive months or more during the year? |
| Wheeze (any) | In the past 12 months have you had wheezing or whistling in your chest? |
| Nocturnal Wheeze | In the past 12 months, how often, on average, has your sleep been disturbed because of wheezing? |
| Wheeze with exertion | In the past 12 months, has your chest sounded wheezy during or after exercise or physical activity? |
| Medications for wheeze | In the past 12 months, have you taken medication, prescribed by a doctor, for wheezing or whistling? |
