# Supplementary material for: Omega-3 fatty acid intake and prevalent respiratory symptoms among U.S. adults with COPD
Source: BMC Pulm Med. 2019 May 21;19:97. doi: 10.1186/s12890-019-0852-4 (PMC6533751; doi:10.1186/s12890-019-0852-4)
Supplement: Supplementary file 3 — Figure S2. Population Distribution of Omega-3 and Omega-6 Fatty Acid Intake in U.S. Adults with COPD. Histograms representing the population distribution of Alpha-linolenic Acid (ALA, omega-3; Panel A, female; Panel B, male) and Eicosapentaenoic Acid + Docosahexaenoic Acid (EPA + DHA, omega-3; Panel C) in grams in U.S. adults with COPD. Vertical red lines represent American Dietetic Association recommended daily intake levels. (DOCX 59 kb) [file 12890_2019_852_MOESM3_ESM.docx]

**(A)**

**(B)**

**(C)**

**Supplemental Figure 2, Additional file 3**: **Population Distribution of Omega-3 and Omega-6 Fatty Acid Intake in U.S. Adults with COPD**
