# Supplementary material for: Omega-3 fatty acid intake and prevalent respiratory symptoms among U.S. adults with COPD
Source: BMC Pulm Med. 2019 May 21;19:97. doi: 10.1186/s12890-019-0852-4 (PMC6533751; doi:10.1186/s12890-019-0852-4)
Supplement: Supplementary file 4 — Table S2. Stratified Characteristics of U.S. Adults with COPD, NHANES 2007–2012. (DOCX 23 kb) [file 12890_2019_852_MOESM4_ESM.docx]

**Supplemental table 2, Additional file 4: Stratified Characteristics of U.S. Adults with COPD, NHANES 2007-2012.**

|  | Stratified Population | | | |
| --- | --- | --- | --- | --- |
| Education | <High School (n=282) | | >High School (n=596) | |
| Smoking Status | Former  (n=133) | Current  (n=149) | Former  (n=323) | Current  (n=273) |
| Demographics |  |  |  |  |
| Age (years) | 66.8 ± 9.7 | 57.6±10.1 | 62.8 ± 7.1 | 57.6 ± 7.4 |
| Male (%) | 69.1 | 68.5 | 64.6 | 58.9 |
| Ethnicity (%)  Non-hispanic White  Other | 72.5  27.5 | 74.4  25.6 | 89.2  10.8 | 87.2  12.7 |
| Pack-Years  BMI, kg/m^2^ | 36.5 ± 46.2  29.5 ± 5.5 | 39.1 ± 31.9  25.7 ± 5.2 | 23.7 ± 19.8  28.5 ± 4.5 | 33.4 ± 19.0  26.0 ± 4.4 |
| Dietary Intake |  |  |  |  |
| Energy (kcal) | 2057 ± 1020 | 2114 ± 731 | 2052 ± 560 | 2198 ± 701 |
| Omega-3 |  |  |  |  |
| EPA+DHA (g) | 0.12±0.29 | 0.09±0.14 | 0.12±0.22 | 0.11±0.19 |
| ALA (g) | 1.63±0.88 | 1.82±1.40 | 1.75±0.79 | 1.63±0.76 |
| Omega-6 |  |  |  |  |
| LA (g) | 15.7±9.0 | 17.5±11.6 | 16.4±6.6 | 16.6±7.7 |
| Lung Function |  |  |  |  |
| FEV_1_ , L | 2.3±0.7 | 2.4±0.7 | 2.5±0.6 | 2.5±0.6 |
| FVC, L | 3.6±1.0 | 3.9±1.0 | 3.9±0.9 | 4.0±0.9 |
| FEV_1_/FVC | 0.63±0.07 | 0.61±0.08 | 0.64±0.05 | 0.62±0.06 |
| Symptom Prevalence (%) | | | | |
| Chronic cough | 9.3 | 33.6. | 9.4 | 27.8 |
| Nocturnal cough | 1.3 | 11.9 | 4.6 | 9.1 |
| Phlegm | 9.4 | 31.6 | 8.5 | 25.6 |
| Wheeze (any) | 16.7 | 28.4 | 14.3 | 27.3 |
| Nocturnal Wheeze | 7.8 | 12.7 | 8.0 | 10.3 |
| Wheeze with exertion | 5.6 | 15.4 | 5.4 | 9.1 |
| Meds for wheeze | 10.1 | 5.3 | 3.9 | 6.0 |

*Mean* ± *SD unless otherwise noted.*

BMI: Body Mass Index; EPA: eicosapentaenoic acid; DHA: docosahexaenoic acid; ALA: alpha-linolenic acid; LA: linoleic acid; FEV_1_: forced expiratory volume in one second; FVC: forced vital capacity.
