# Supplementary material for: Omega-3 fatty acid intake and prevalent respiratory symptoms among U.S. adults with COPD
Source: BMC Pulm Med. 2019 May 21;19:97. doi: 10.1186/s12890-019-0852-4 (PMC6533751; doi:10.1186/s12890-019-0852-4)
Supplement: Supplementary file 5 — Table S3. Intake of omega-3 and -6 fatty acids in U.S. adults with COPD, NHANES 2007–2012. Average fatty acid intake in US/ Individuals with COPD stratified by personal characteristic or exposure. (DOCX 28 kb) [file 12890_2019_852_MOESM5_ESM.docx]

**Supplemental Table 3, Additional file 5: Intake of omega-3 and -6 fatty acids in U.S. adults with COPD, NHANES 2007-2012.**

|  | Sample  (n) | Population* (n) | Omega-6: LA | | Omega-3: ALA | | Omega-3: EPA+DHA | |
| --- | --- | --- | --- | --- | --- | --- | --- | --- |
|  |  |  | (g) | p-value | (g) | p-value | (g) | p-value |
| Age (years) |  |  |  |  |  |  |  |  |
| 40-49 | 106 | 1,838,621 | 18.36 ± 9.35 | 0.15 | 1.84±1.16 | 0.74 | 0.06±0.11 | **0.001** |
| 50-59 | 193 | 4,106,040 | 17.44 ± 6.69 |  | 1.75±0.65 |  | 0.14±0.23 |  |
| 60-69 | 314 | 4,230,628 | 15.69 ± 7.28 |  | 1.64±0.78 |  | 0.11±0.18 |  |
| 70-79 | 265 | 2,546,322 | 15.22 ± 8.71 |  | 1.67±1.10 |  | 0.12±0.23 |  |
| Gender (%) |  |  |  |  |  |  |  |  |
| Male | 624 | 8,075,560 | 17.80±9.06 | **<0.001** | 1.82±1.00 | **0.01** | 0.12±0.26 | 0.15 |
| Female | 254 | 4,646,051 | 14.35±5.74 |  | 1.52±0.66 |  | 0.10±0.13 |  |
| Ethnicity (%) |  |  |  |  |  |  |  |  |
| Non-Hispanic White | 565 | 10,800,000 | 16.77±7.00 | 0.10 | 1.73±0.77 | 0.25 | 0.11±0.18 | 0.24 |
| Other | 313 | 1,890,659 | 15.21±11.65 |  | 1.60±1.38 |  | 0.13±0.31 |  |
| Education |  |  |  |  |  |  |  |  |
| <High School | 282 | 2,730,216 | 16.77±11.04 | 0.85 | 1.75±1.28 | 0.81 | 0.10±0.20 | 0.56 |
| >High School | 596 | 9,991,395 | 16.48±7.14 |  | 1.70±0.78 |  | 0.12±0.21 |  |
| BMI, kg/m^2^ |  |  |  |  |  |  |  |  |
| Underweight | 26 | 327,007 | 14.94±9.33 | 0.88 | 1.40±0.81 | 0.16 | 0.12±0.19 | 0.68 |
| Normal | 294 | 4,365,022 | 16.35±7.85 |  | 1.65±0.81 |  | 0.10±0.18 |  |
| Overweight | 312 | 4,687,442 | 16.86±8.79 |  | 1.79±1.02 |  | 0.13±0.26 |  |
| Obese | 241 | 3,295,397 | 16.52±6.52 |  | 1.71±0.75 |  | 0.10±0.16 |  |
| Smoking status |  |  |  |  |  |  |  |  |
| Former | 456 | 6,515,828 | 16.29±7.12 | 0.46 | 1.73±0.83 | 0.55 | 0.12±0.23 | 0.46 |
| Current | 422 | 6,205,783 | 16.80±8.78 |  | 1.68±0.94 |  | 0.11±0.18 |  |
| Pack years^ɵ^ |  |  |  |  |  |  |  |  |
| Low | 419 | 6,161,650 | 17.16±8.39 | 0.27 | 1.79±1.00 | 0.20 | 0.11±0.23 | 0.68 |
| High | 427 | 6,144,228 | 15.95±7.64 |  | 1.63±0.74 |  | 0.11±0.19 |  |

*Mean* ± *SD unless otherwise noted.*

*Population estimates based on use of NHANES sampling weights.

^ɵ^dichotomized at median

BMI: Body Mass Index; EPA: eicosapentaenoic acid; DHA: docosahexaenoic acid; ALA: alpha-linolenic acid. LA: linoleic acid.
